# Translating preventive chemotherapy prevalence thresholds for *Schistosoma mansoni* from the Kato-Katz technique into the point-of-care circulating cathodic antigen diagnostic test

**DOI:** 10.1371/journal.pntd.0006941

**Published:** 2018-12-14

**Authors:** Oliver Bärenbold, Amadou Garba, Daniel G. Colley, Fiona M. Fleming, Ayat A. Haggag, Reda M. R. Ramzy, Rufin K. Assaré, Edridah M. Tukahebwa, Jean B. Mbonigaba, Victor Bucumi, Biruck Kebede, Makoy S. Yibi, Aboulaye Meité, Jean T. Coulibaly, Eliézer K. N’Goran, Louis-Albert Tchuem Tchuenté, Pauline Mwinzi, Jürg Utzinger, Penelope Vounatsou

**Affiliations:** 1 Swiss Tropical and Public Health Institute, Basel, Switzerland; 2 University of Basel, Basel, Switzerland; 3 Department of Control of Neglected Tropical Diseases, World Health Organization, Geneva, Switzerland; 4 Center for Tropical and Emerging Global Diseases and Department of Microbiology, University of Georgia, Athens, GA, United States of America; 5 Schistosomiasis Control Initiative, Imperial College, London, United Kingdom; 6 Ministry of Health and Population, Cairo, Egypt; 7 National Nutrition Institute, General Organisation for Teaching Hospitals and Institutes, Cairo, Egypt; 8 Centre Suisse de Recherches Scientifiques en Côte d’Ivoire, Abidjan, Côte d’Ivoire; 9 Unité de Formation et de Recherche Biosciences, Université Félix Houphouët-Boigny, Abidjan, Côte d’Ivoire; 10 Vector Control Division, Ministry of Health, Kampala, Uganda; 11 Ministry of Health, Kigali, Rwanda; 12 Programme National Intégré de Lutte contre les Maladies Tropicales Négligées et la Cécité au Burundi, Bujumbura, Burundi; 13 Ministry of Health, Addis Ababa, Ethiopia; 14 Neglected Tropical Disease Department, Ministry of Health, Juba, South Sudan; 15 Programme National de Lutte contre les Maladies Tropicales Négligées à Chimiothérapie Préventive, Ministère de la Santé et de l’Hygiène Publique, Abidjan, Côte d’Ivoire; 16 Laboratory of Parasitology and Ecology, University of Yaoundé I, Yaoundé, Cameroon; 17 Centre for Schistosomiasis and Parasitology, Yaoundé, Cameroon; 18 Centre for Global Health Research, Kenya Medical Research Institute, Nairobi, Kenya; Institut de Recherche pour le Développement, BENIN

## Abstract

**Background:**

Intervention guidelines against *Schistosoma mansoni* are based on the Kato-Katz technique. However, Kato-Katz thick smears show low sensitivity, especially for light-intensity infections. The point-of-care circulating cathodic antigen (POC-CCA) is a promising rapid diagnostic test detecting antigen output of living worms in urine and results are reported as trace, 1+, 2+, and 3+. The use of POC-CCA for schistosomiasis mapping, control, and surveillance requires translation of the Kato-Katz prevalence thresholds into POC-CCA relative treatment cut-offs. Furthermore, the infection status of egg-negative but antigen-positive individuals and the intensity-dependent sensitivity of POC-CCA should be estimated to determine its suitability for verification of disease elimination efforts.

**Methodology:**

We used data from settings in Africa and the Americas characterized by a wide range of *S. mansoni* endemicity. We estimated infection intensity-dependent sensitivity and specificity of each test at the unit of the individual, using a hierarchical Bayesian egg-count model that removes the need to define a ‘gold’ standard applied to data with multiple Kato-Katz thick smears and POC-CCA urine cassette tests. A simulation study was carried out based on the model estimates to assess the relation of the two diagnostic tests for different endemicity scenarios.

**Principal findings:**

POC-CCA showed high specificity (> 95%), and high sensitivity (> 95%) for moderate and heavy infection intensities, and moderate sensitivity (> 75%) for light infection intensities, and even for egg-negative but antigen-positive infections. A 10% duplicate slide Kato-Katz thick smear prevalence corresponded to a 15–40% prevalence of ≥ trace-positive POC-CCA, and 10–20% prevalence of ≥ 1+ POC-CCA. The prevalence of ≥ 2+ POC-CCA corresponded directly to single slide Kato-Katz prevalence for all prevalence levels.

**Conclusions/significance:**

The moderate sensitivity of POC-CCA, even for very light *S. mansoni* infections where the sensitivity of Kato-Katz is very low, and the identified relationship between Kato-Katz and POC-CCA prevalence thresholds render the latter diagnostic tool useful for surveillance and initial estimation of elimination of *S. mansoni*. For prevalence below 10% based on a duplicate slide Kato-Katz thick smear, we suggest using POC-CCA including trace results to evaluate treatment needs and propose new intervention thresholds that need to be validated in different settings.

## Author summary

The World Health Organization (WHO) has defined goals for schistosomiasis morbidity control to be reached by 2025 that are based on preventive chemotherapy. Intervention thresholds for *Schistosoma mansoni* are currently defined for prevalence measured by stool microscopy using the Kato-Katz technique. However, the Kato-Katz technique shows low sensitivity, particularly for the detection of light-intensity infections. Replacing it with the semi-quantitative point-of-care circulating cathodic antigen (POC-CCA) urine cassette test requires translation of the thresholds and precise characterization of the diagnostic sensitivity and specificity. In this study, we applied a novel egg-count model to a suite of data obtained from different settings in Africa and the Americas with diverse endemicity levels. We used a simulation study to infer on the relation between Kato-Katz and POC-CCA prevalence. Based on our study, we were able to provide recommendations for POC-CCA thresholds taking into account semi-quantitative results of the test. We found that a *S. mansoni* prevalence of 10% based on duplicate slide Kato-Katz thick smear is equivalent to 15–40% POC-CCA prevalence when trace results are considered positive and to 10–20% POC-CCA prevalence when trace results are considered negative. Our results have important bearings for mapping, control, surveillance, and verification of elimination of intestinal schistosomiasis.

## Introduction

Schistosomiasis is a neglected tropical disease (NTD) caused by parasitic flatworms of the genus *Schistosoma* that require freshwater snails as intermediate hosts to complete their life cycle [1]. Currently, around 250 million people are infected, primarily school-aged children in rural areas of sub-Saharan Africa, and, to a lesser extent in Southeast Asia, and the Americas, causing an estimated 2.1 million disability-adjusted life years (DALYs) in 2016 [2, 3]. There are six species infecting humans, of which *Schistosoma haematobium*, *S. japonicum*, and *S. mansoni* are the most common ones [1].

This study discusses the diagnosis of *S. mansoni* whose adult stage live in pairs within the mesenteric venules. Experiments in mice showed that female worms shed in the order of several hundred eggs daily with a large variability. Eggs are either trapped in the intestines and liver causing inflammatory reactions, or are excreted through feces continuing the life cycle [4]. Morbidity, among others, includes anemia, growth stunting, impaired cognition, increased susceptibility to other infections (e.g., HIV), and severe pathologies of the liver and spleen [1, 5–7].

The World Health Organization (WHO) has articulated a road-map for morbidity control, and elimination of the disease in suitable settings, by 2025 based on preventive chemotherapy using praziquantel administered to school-aged children and other high-risk communities, control of intermediate host snails, and behavioral and environmental changes [8, 9]. To achieve these goals, intervention guidelines were set that define the communities requiring preventive chemotherapy and treatment frequency. Enacting these guidelines requires estimates of the disease prevalence, which are obtained from survey data. Transmission levels may vary over short geographic scales (2–5 km), and thus, having the ability to sample large numbers of communities with rapid, low cost, and sensitive tests are critical for efficient and effective disease control [10–12].

Infections with *S. mansoni* can be diagnosed using various techniques to detect eggs in stool. The most widely used method is the Kato-Katz technique based on a thick smear, usually with a volume equivalent to 41.7 mg [13]. Of note, the Kato-Katz technique has been used to define intervention thresholds for preventive chemotherapy [14]. While fecal egg detection techniques have generally high specificity, they suffer from low sensitivity, especially for light-intensity infections, which lead to underestimation of community prevalence and burden [15, 16]. To reduce the diagnostic error, repeated sampling of stool and preparation of multiple Kato-Katz thick smears from a single stool specimen is recommended, which is impractical and expensive for disease control purposes [17]. Furthermore, Kato-Katz detects only infections with mature, egg-shedding worms. To overcome these shortcomings, novel techniques that are not based on egg detection are required.

A promising candidate is a test that detects a specific antigen in urine; the point-of-care circulating cathodic antigen (POC-CCA) [18]. Diagnosis takes about 20 min and the outcome is reported semi-quantitatively; namely, trace, 1+, 2+, and 3+, although interpretation may depend on the laboratory technician. Systematic reviews estimate the sensitivity to be around 90%. POC-CCA classifies individuals as positive that were classified as negative by Kato-Katz due to its low sensitivity for light infection intensities. Hence, the specificity of POC-CCA is underestimated when Kato-Katz is considered as diagnostic ‘gold’ standard [19, 20]. Colley et al. performed regression analysis to estimate the relation between POC-CCA and single Kato-Katz thick smears and indicated the need for further investigation to determine how it depends on different sampling schemes and levels of endemicity [21]. Converting Kato-Katz prevalence thresholds from existing treatment guidelines into POC-CCA analogues requires information on the variation of the sensitivity of POC-CCA with infection intensity. Moreover, the prevalence of egg-negative but antigen- or worm-positive infections and its influence on the prevalence measured by POC-CCA in comparison with Kato-Katz testing has to be evaluated.

In this study, we addressed the aforementioned issues using pairs of data with simultaneous testing of fecal samples by Kato-Katz and urine samples by POC-CCA from various settings in Africa and the Americas, characterized by a wide range of endemicity levels. We developed a model which infers on the infection intensity-dependent sensitivity profile of POC-CCA for semi-quantitative test results without using an artificial ‘gold’ standard. Additionally, we estimated the specificity of POC-CCA and the prevalence of egg-negative/antigen-positive infections. Model outputs were employed in a simulation study to obtain insights on the relation between measured Kato-Katz and POC-CCA prevalence, assuming a range of scenarios with various infection intensities and prevalence of egg-negative infections. Our findings translate Kato-Katz based *S. mansoni* prevalence thresholds put forth in the current WHO intervention guidelines into POC-CCA prevalence and enable the latter diagnostic approach for mapping, disease control, and surveillance.

## Materials and methods

### Ethics statement

The data included in this study were published elsewhere or are currently in the process of being published [21–31]. Hence, ethics approval, written informed consent procedures, and treatment of infected individuals are given in the respective studies where the original data were or are being published.

### Data

We analyzed a suite of 30 datasets with simultaneous Kato-Katz and POC-CCA results available at individual level. A description of the data related to Kato-Katz and POC-CCA results is given in Tables 1 and 2, respectively. The data were grouped in four categories depending on their characteristics. In particular, a number of datasets (group 1) from Cameroon, Côte d’Ivoire, Ethiopia, Kenya, and Uganda have at least duplicate Kato-Katz readings on two different days and at least one POC-CCA urine cassette test result. These datasets were used in the most complex egg-count model to estimate infection intensity-dependent sensitivity of both diagnostic methods and can be found in S2 Table. Data from Ecuador and Ethiopia with binary Kato-Katz results (i.e., egg-positive or egg-negative; group 2) were collected at locations known to be non-endemic and therefore include information about the specificity of POC-CCA. Datasets from Burundi, Côte d’Ivoire, Rwanda, and South Sudan, with only duplicate Kato-Katz slides from one stool sample (group 3) did not provide information on the day-to-day variation. Datasets from Egypt (group 4) with single Kato-Katz readings of binary outcome were collected from locations with very low transmission. Data from groups 3 and 4 were used in the simulation study.

**Table 1:** Summary of the Kato-Katz results of the databases.

Country	Location	Date (year)	Age range (years)	z_d_ ^1^	z_s_ ^2^	N_KK_	P. KK (%)	*μ* KK (EPG)	geom. *μ* (EPG)	P 1 KK (%)
**Group 1**										
Cameroon	Makenene [24]	2010	6-16	3	3	251	71.7	161	43.3	41.7 (34.8, 48.7)
Cameroon	Njombe [24]	2010	8-16	3	3	245	63.3	173	27.5	30.6 (26.9, 34.3)
Cameroon	Yaounde [24]	2010	7-14	3	3	233	27.9	235	40.9	16.5 (12.3, 20.7)
Côte d’Ivoire	Man [25]	2016	9-13	2	3	695	6.5	72	22.0	3.8 (2.6, 4.9)
Côte d’Ivoire	1 [26]	2011	0.2-5.5	2	2	109	25.7	90	37.0	16.5 (12.3, 20.8)
Côte d’Ivoire	2 [26]	2011	0.2-5.5	2	2	133	21.1	122	30.8	11.7 (9.1, 14.3)
Côte d’Ivoire	1 [22]	2010	8-12	3	3	170	91.7	525	248.0	70.2 (62.4, 78.1)
Côte d’Ivoire	2 [22]	2010	8-12	3	3	130	53.1	116	36.8	24.5 (14.8, 34.3)
Côte d’Ivoire	3 [22]	2010	8-12	3	3	146	32.9	50	8.5	8.3 (3.1, 13.5)
Ethiopia	Harbu [27]	2010	8-12	3	2	300	57.0	69	31.0	33.1 (24.8, 41.4)
Ethiopia	Jiga [27]	2010	8-12	3	2	320	49.4	153	70.9	35.8 (32.1, 39.5)
Kenya	[23]	2007	1-15	3	2	1,845	22.1	106	32.1	11.4 (7.7, 15.2)
Uganda	1 [28]	2010	7-13	3	2	100	55.0	240	34.2	29.3 (24.0, 34.6)
Uganda	2 [28]	2010	7-13	3	2	100	54.0	122	33.3	29.8 (23.6, 36.1)
Uganda	3 [28]	2010	7-13	3	2	100	31.0	37	19.8	14.9 (9.7, 20.1)
Uganda	4 [28]	2010	7-13	3	2	100	35.0	247	58.0	21.1 (16.8, 25.4)
Uganda	5 [28]	2010	7-13	3	2	100	12.0	58	28.4	6.8 (3.8, 9.8)
Uganda	Baseline	2013	6-16	3	2	775	6.3	48	22.0	3.1 (1.5, 4.7)
Uganda	Follow-up	2015	6-16	3	2	659	4.2	68	33.5	2.7 (1.5, 3.9)
Uganda	Mapping	2013	9-14	3	2	711	3.8	182	26.9	1.8 (1.0, 2.6)
**Group 2**										
Ecuador	[31]	2014	6-16	1	1	144	0	-	-	-
Ethiopia	[21]	2010	8-12	1	1	100	0	-	-	-
**Group 3**										
Burundi	[30]	2014	12-16	1	2	8,482	1.5	56	34.4	1.2 (1.1, 1.3)
Côte d’Ivoire	All		6-15	1	2	11,449	8.0	267	80.3	6.1 (5.6, 6.6)
Rwanda	All	2014		1	2	8,695	2.0	84	52.0	1.7 (1.5, 2.0)
South Sudan	All		10-14	1	2	5,649	7.1	128	54.1	5.7 (5.1, 6.3)
**Group 4**										
Egypt	Gov 1 [29]	2016	6-15	1	1	3,000	3.5	-	-	-
Egypt	Gov 2 [29]	2016	6-15	1	1	5,000	1.7	-	-	-
Egypt	Gov 3 [29]	2016	6-15	1	1	2,946	0.1	-	-	-
Egypt	Gov 4 [29]	2016	6-15	1	1	974	0.4	-	-	-
Egypt	Gov 5 [29]	2016	6-15	1	1	2,997	0.1	-	-	-

^1^ *z*_*d*_ is the number of stool specimens taken on different days

^2^ *z*_*s*_ is the number of Kato-Katz thick smears prepared by a single stool specimens

**Table 2:** Summary of POC-CCA results of databases employed for the current modeling study to translate Kato-Katz to POC-CCA prevalence intervention thresholds.

Country	Location	z_r_ ^1^	N_CCA_	P. CCA Tr+^2^	(%)	Tr−^3^		2 − 3+^4^	
**Group 1**									
Cameroon	Makunene	3	270	85.2	75.9 (68.2, 83.7)	60.4	52.1 (48.7, 55.5)	-	-
Cameroon	Njombe	3	270	87.8	75.2 (73.0, 77.4)	55.9	43.1 (34.4, 51.7)	-	-
Cameroon	Yaounde	3	237	72.1	50.8 (45.7, 55.9)	24.1	17.2 (13.6, 20.7)	-	-
Côte d’Ivoire	Man	1	700	32.7	-	20.4	-	-	-
Côte d’Ivoire	1	2	109	81.7	67.0 (61.8, 72.2)	44.0	35.8 (22.8, 48.8)	27.5	22.0 (66.7, 76.4)
Côte d’Ivoire	2	2	109	72.2	58.3 (50.8, 65.7)	45.9	32.0 (24.5, 39.4)	24.1	17.7 (8.1, 27.2)
Côte d’Ivoire	1	3	170	-	-	86.5	83.3 (81.5, 85.1)	77.6	69.4 (66.3, 72.5)
Côte d’Ivoire	2	3	130	-	-	51.5	40.5 (27.4, 53.6)	23.1	15.9 (13.5, 18.2)
Côte d’Ivoire	3	3	146	-	-	34.2	23.1 (22.3, 23.9)	6.8	5.0 (3.4, 6.6)
Ethiopia	Harbu	3	300	80.0	71.2 (65.0, 77.4)	-	-	-	-
Ethiopia	Jiga	3	320	62.5	59.6 (58.6, 60.5)	-	-	-	-
Kenya		3	1,845	74.4	53.3 (51.0, 55.5)	-	-	11.6	7.7 (6.8, 8.6)
Uganda	1	1	100	70.0	-	52.0	-	28.0	-
Uganda	2	1	100	74.0	-	56.0	-	22.0	-
Uganda	3	1	100	65.0	-	52.0	-	20.0	-
Uganda	4	1	100	56.0	-	46.0	-	20.0	-
Uganda	5	1	100	48.0	-	35.0	-	7.0	-
Uganda	Base	3	775	33.7	21.0 (19.0, 22.9)	13.4	8.5 (6.5, 10.5)	5.2	3.1 (1.0, 5.3)
Uganda	F1	3	659	37.0	21.2 (13.9, 28.6)	19.0	11.4 (8.7, 14.2)	2.4	1.6 (0.7, 2.4)
Uganda	Mapping	3	711	19.0	11.2 (10.3, 12.2)	7.3	4.6 (3.8, 5.5)	2.0	1.6 (1.3, 1.8)
**Group 2**									
Ecuador		1	144	0	-	-	-	-	-
Ethiopia		1	100	1	-	-	-	-	-
**Group 3**									
Burundi		1	8,482	41.3	-	10.9	-	-	-
Côte d’Ivoire	All	1	11,453	20.9	-	-	-	-	-
Rwanda	All	1	8,695	37.5	-	8.6	-	-	-
South Sudan	All	1	5,649	41.5	-	-	-	-	-
**Group 4**									
Egypt	Gov 1	1	3,000	17.6	-	-	-	-	-
Egypt	Gov 2	1	5,000	9.4	-	-	-	-	-
Egypt	Gov 3	1	2,946	4.6	-	-	-	-	-
Egypt	Gov 4	1	974	9.7	-	-	-	-	-
Egypt	Gov 5	1	2,997	12.3	-	-	-	-	-

^1^ *z*_*r*_ is the number of POC-CCA tests performed on different days

^2^ **Tr**+ is the prevalence when trace, 1+, 2+, and 3+ results are considered positive

^3^ **Tr**− is the prevalence when 1+, 2+, and 3+ results are considered positive

^4^ **2** − **3**+ is the prevalence when 2+ and 3+ results are considered positive

### Statistical model

The Kato-Katz results were available either as binary or egg-count measurements. Hence, we developed separate but interlinked Bayesian hierarchical models for each type of data to include all available information in the evaluation of POC-CCA.

#### Egg-count model

We extended our previous work modeling Kato-Katz egg-count data without the need of an artificial ‘gold’ standard to include POC-CCA results and estimated the POC-CCA infection intensity-dependent sensitivity [16]. Similar models have recently been applied by Bottomley et al. (2016) [32] to model the diagnostic sensitivity for *Onchocerca volvulus*, and by Prada et al. (2018) [33] to compare POC-CCA to Kato-Katz diagnostics for *S. mansoni*.

Let $Y_{jids}^{KK}$ be the Kato-Katz egg count of individual *i* in study *j* on day *d* when reading slide *s*, and $Y_{jirk}^{CCA}$ the binary result of the POC-CCA reading based on proxy *k* from individual *i* in study *j* on repeat *r*. Three proxies have been considered to convert the semi-quantitative reading into a binary outcome and estimate the equivalent Kato-Katz prevalence; i.e., *k* = 1 treats all positive results, including trace, as positive; *k* = 2 takes 1+, 2+, and 3+ results as positive; and *k* = 3 groups only 2+ and 3+ results as positive. We assumed that the population in study *j* consists of a proportion of infected individuals that are egg-positive *p*_*j*_ with individual disease status *D*_*ji*_ = 2, the proportion of antigen-positive individuals that is egg-negative *l*_*j*_ with disease status *D*_*ji*_ = 1, and the uninfected individuals *D*_*ji*_ = 0 with proportion 1 − *p*_*j*_ − *l*_*j*_. We assumed that the diagnostic results conditional on the disease status and infection intensity are independent. This means that we assume that the sensitivity of a diagnostic test for an individual is fully determined by the egg-density in stool and it is independent of other individual level factors. Let **Y**_*ji*_ = (*Y*_*ji*11_, *Y*_*ji*12_,…, *Y*_*jids*_)^*T*^.

$$\begin{array}{c} \begin{array}{cc} P(Y_{jik}^{KK},Y_{ji}^{CCA}) & =\sum_{Z=0}^{2}P\left(Y_{jik}^{KK}|D_{ji}=Z\right)\cdot P\left(Y_{ji}^{CCA}|D_{ji}=Z\right)\cdot P\left(D_{ji}=Z\right) \end{array} \end{array}$$ (1)

For the non-infected individuals (*D*_*ji*_ = 0), with infection intensity λ_*ji*_ = 0, the egg count $Y_{jids}^{KK}$ is modeled by a negative binomial distribution, and the POC-CCA binary results $Y_{jirk}^{CCA}$ by a binomial distribution, as follows:

$$\begin{array}{c} \begin{array}{cc} P(Y_{ji}^{KK}|D_{ji}=0) & \equiv \prod_{d,s}NB\left(\mu =\gamma_{1}\cdot \left({\left(c^{KK}\right)}^{-1/\gamma_{1}}-1\right),\gamma_{1}\right)\\ P(Y_{jik}^{CCA}|D_{ji}=0) & \equiv \prod_{r}Be\left(1-c_{k}^{CCA}\right) \end{array} \end{array}$$ (2)

where *c*^*KK*^ is the specificity of the Kato-Katz technique, which is assumed to be the same for each study, *γ*_1_ is the dispersion parameter of the false positives, and $c_{k}^{CCA}$ is the specificity of the POC-CCA test for proxy *k*.

For egg-positive individuals (*D*_*ji*_ = 2), we assumed that each individual has a mean infection intensity λ_*ji*_ that is related to the number of worm pairs. Daily variations in egg-output are described using a mean egg output λ_*jid*_ for day *d*. We assumed that the Kato-Katz result for the infected individuals on day *d* and slide *s* are independent, conditional on the infection intensity λ_*ji*_ for individual *i* and on the day-to-day variation *ϵ*_*jid*_, and modeled by a negative binomial distribution.

$$\begin{array}{c} \begin{array}{cc} P(Y_{ji}^{KK}|D_{ji}=2) & \equiv \prod_{d,s}NB\left(\lambda_{jid}+\mu_{min},\gamma_{2j}\right)\\ \text{log}\left(\lambda_{jid}\right)|\lambda_{ji},\epsilon_{jid} & =\text{log}\left(\lambda_{ji}\right)+\epsilon_{jid}\\ \epsilon_{jid} & ∼N(0,\sigma_{j}^{2})\\ \lambda_{ji} & ∼\text{Gamma}(\mu_{f,j}\cdot \alpha_{j},\alpha_{j}) \end{array} \end{array}$$ (3)

*α*_*j*_ models the variation within the population of infected individuals in study *j*, $\sigma_{j}^{2}$ captures the extent of the day-to-day variation *ϵ*_*jid*_, *γ*_2*j*_ takes into account the non-random distribution of eggs within a sample, *μ*_*min*_ is the minimum possible infection intensity corresponding to one pair of worms, and *μ*_*f*,*j*_ corresponds to the mean infection intensity of infected individuals in study *j*. False-negatives are included in the model as repeated zero measurements, e.g., the sensitivity for a single Kato-Katz reading becomes

$$\begin{array}{c} s_{jid}^{KK}=1-\text{NB}\left(Y_{jids}^{KK}=0;\lambda_{jid},\gamma_{2j}\right)=1-(\frac{\gamma_{2j}}{\lambda_{jid}+\gamma_{2j}}{)}^{\gamma_{2j}} \end{array}$$ (4)

$Y_{jirk}^{CCA}$ is modeled by a binomial distribution with the sensitivity of POC-CCA, $s_{jik}^{CCA}$, dependent on the infection intensity of the individual, given by

$$\begin{array}{c} \begin{array}{cc} P(Y_{jirk}^{CCA}|D_{ji}=2) & \equiv Be(s_{jik}^{CCA})\\ s_{jik}^{CCA}\left(\lambda_{ji}\right) & ={\mathrm{logit}}^{-1}\left(a_{0kj}+a_{1kj}\cdot \sqrt{\lambda_{ji}}\right)\cdot a_{2kj} \end{array} \end{array}$$ (5)

*a*_0*kj*_ determines the sensitivity of a very light infection intensity in study *j*, *a*_1*kj*_ describes the dependence of the sensitivity on the infection intensity, and *a*_2*kj*_ determines the limit of the sensitivity for severe infections.

The infected but non egg-shedding individuals *D*_*ji*_ = 1 are modeled similar to the uninfected ones but with differing parameters for POC-CCA.

$$\begin{array}{c} \begin{array}{cc} P(Y_{jids}^{KK}|D_{ji}=1) & \equiv NB(\mu =\gamma_{1}\cdot \left(c_{KK}^{-1/\gamma_{1}}-1\right),\gamma_{1})\\ P(Y_{jirk}^{CCA}|D_{ji}=1) & \equiv Be(s_{jik}^{CCA}\left(\lambda_{ji}=0\right)) \end{array} \end{array}$$ (6)

where $s_{jik}^{CCA}\left(\lambda_{ji}=0\right)$ is the sensitivity of POC-CCA for egg-negative infections, which is considered to be the same as for egg shedding infections with an infection intensity equal to zero eggs per gram of stool (EPG). Kato-Katz readings are assumed to have the same distribution as for non-infected individuals.

The parameters that are related to the biology of the worms, to transmission behavior or to the diagnostic technique and expected to be related between studies, i.e., the within-population variation *α*_*j*_, the day-to-day variation $\sigma_{j}^{2}$, the slide-to-slide variation *γ*_*j*_, the parameters determining the sensitivity of POC-CCA, namely *a*_0*kj*_, *a*_1*kj*_, and *a*_2*kj*_, were partially pooled, using a common mean and a normally distributed random effect on the log scale.

#### Binary latent class model

Binary POC-CCA data from locations with zero Kato-Katz positive individuals were included to extract information about the specificity of POC-CCA. The model was formulated as follows:

$$\begin{array}{c} Y_{ikr}^{CCA}∼Be\left(s_{jik}^{CCA}\left(\lambda_{ji}=0\right)\pi +\left(1-c_{k}^{CCA}\right)\left(1-\pi \right)\right) \end{array}$$ (7)

where $s_{jik}^{CCA}\left(\lambda_{ji}=0\right)$ is the sensitivity of POC-CCA for egg-negative infections, used as a lower boundary, and *π* the prevalence in the given setting.

#### Implementation details

The aforementioned model was fitted using Markov chain Monte Carlo (MCMC) simulations in Stan version 2.16.2 (Stan Development Team; http://mc-stan.org) with 25 chains consisting of 500 warm-up and 500 sampling steps [34]. Subsequent analyses and simulations were performed using R version 3.4.1 (The R Foundation for Statistical Computing; Vienna, Austria) and RStudio version 1.0.143 (RStudio, Inc.; Boston, United States of America).

An informative Beta prior was considered for the specificity of the Kato-Katz technique *c*^*KK*^ with mean 0.98 and standard deviation (SD) 0.01. We assumed a rather informative truncated normal prior with mean 0.03 and SD 0.01 for the infection intensity of one pair of worms *μ*_*min*_. The mean was chosen by assuming an average egg output of a pair of worms in the order of 100 eggs per day, multiplied with the ratio between the weight of a Kato-Katz sample of 41.7 mg and a daily production of feces of 150 g which corresponds to about 0.03 eggs per sample or 0.72 EPG for *S. mansoni* [4]. For the dispersion of eggs detected in a non-infected individual *γ*_1_, we assumed a truncated normal prior with mean and variance of 1 to ensure that the typical false positive has a low egg count. The mean infection intensity of an infected individual in study *j* was calculated as follows:

$$\begin{array}{c} \mu_{j}=\mu_{f,j}\cdot e^{\sigma_{j}^{2}/2}+\mu_{min} \end{array}$$ (8)

Semi-informative or non-informative priors were adopted for the rest of the parameters: a uniform distribution between 0 and 1 for $c_{k}^{CCA}$, gamma distributions for *σ*_*j*_, *α*_*j*_, and *γ*_2*j*_ with mean 1 and SD 1, gamma distribution with mean 10 and SD 10 for *μ*_*f*,*j*_, normal distribution for *a*_0_ with mean 0 and SD 2, gamma distribution with mean 5 and SD 5 for *a*_1_, normal distribution with mean 2 and SD 2 on the logit of *a*_2_, and uniform priors between 0 and 1 for all prevalence parameters.

### Simulation study

Using the parameter estimates described in our statistical model, we simulated single and duplicate slide Kato-Katz thick smears and POC-CCA prevalences under 84 different scenarios. The simulations were carried out with a large number of individuals per scenario to avoid variations due to sampling. We assumed a wide range of egg-positive infections (i.e., 5%, 10%, 20%, 30%, 50%, and 70%) to mimic different endemicity scenarios. Prevalence of egg-negative infections was varied independently from the egg-positive prevalence and assumed to be 5%, 10%, 20%, and 30%. Mean infection intensity of an egg-positive infected individual was set to 50 EPG, 100 EPG, 200 EPG, and 400 EPG.

## Results

### Characterization of POC-CCA

Our model estimates the sensitivity and specificity in relation to the ‘true’ prevalence of infection in the population. This avoids the use of a ‘gold’ standard, which does not exist. Posterior estimates of parameters that influence the sensitivity and specificity can be found in Table A in S1 Table.

**Fig 1:**
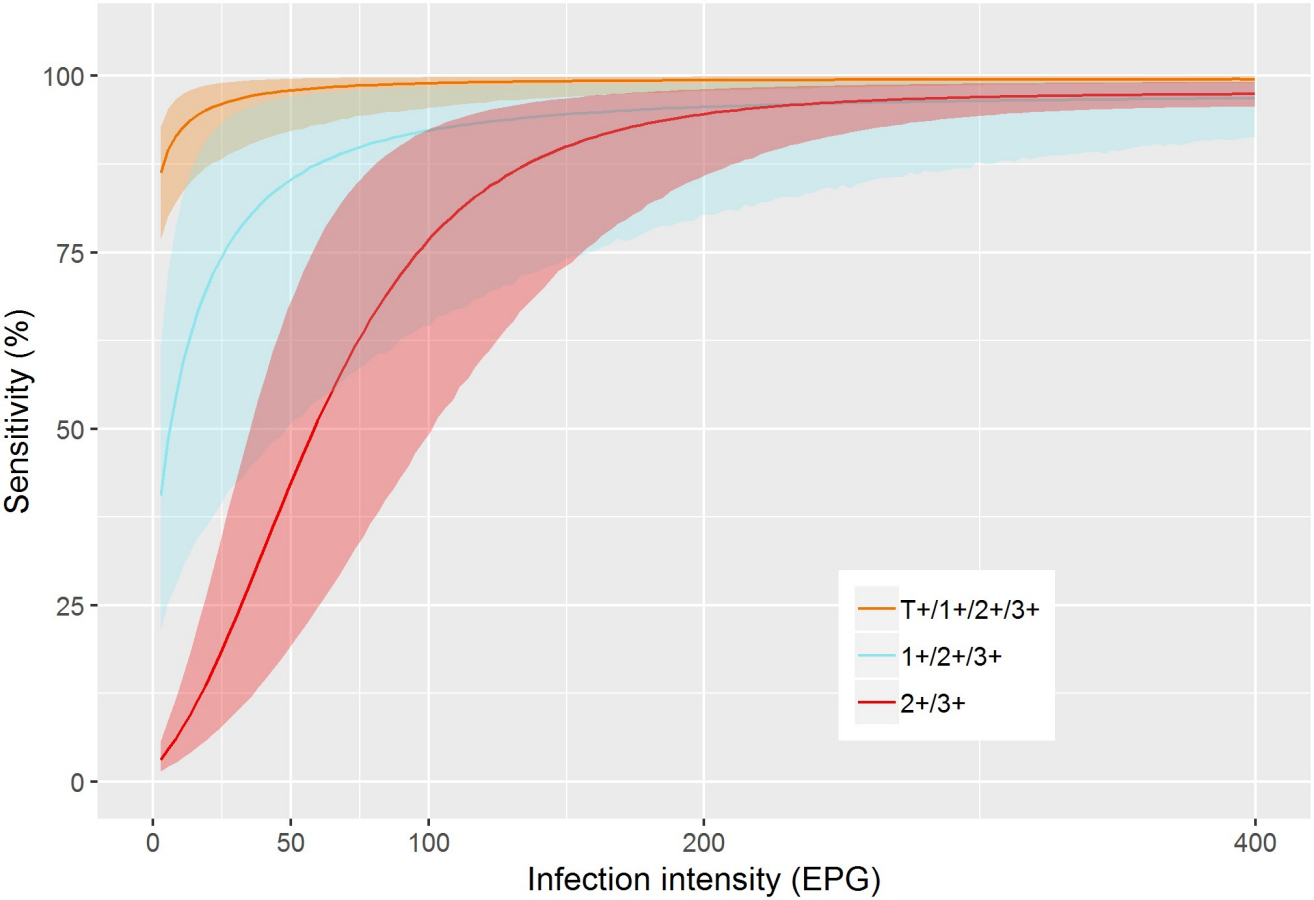
Model-based estimate of the infection intensity-dependent sensitivity of POC-CCA.

Based on these parameters, Fig 1 shows the infection-dependent sensitivity of POC-CCA. All three binary proxies of POC-CCA indicate a very high sensitivity for moderate and heavy infections (≥ 100 EPG). The sensitivity for light infections differ between proxies: while including trace results as positives enables detection of very light infections, considering 2+ and 3+ results as positives leads to missing of many infected individuals. The uncertainty in the estimated sensitivity is largest in the proxy considering trace results as negative (1+/2+/3+).

Table 3 shows the specificity and sensitivity estimates for egg-negative infections. POC-CCA revealed very high sensitivity even for light infection intensities when trace results were interpreted as positive. Furthermore, sensitivity was above 60% for non-egg-shedding but antigen-positive individuals that apparently harbor worms. The proxy 2+ and 3+ revealed low sensitivity for egg-negative/antigen-positive individuals. Specificity is over 95% irrespective of the chosen proxy.

**Table 3:** Model-based estimates of the specificity and sensitivity for egg-negative infections of POC-CCA.

	Specificity	Sensitivity–egg-negative/antigen-positive
T/1+/2+/3+	0.96 (0.95, 0.97)	0.75 (0.65, 0.84)
1+/2+/3+	1.00 (0.99, 1.00)	0.23 (0.12, 0.39)
2+/3+	1.00 (0.99, 1.00)	0.01 (0.01, 0.02)

**Fig 2:**
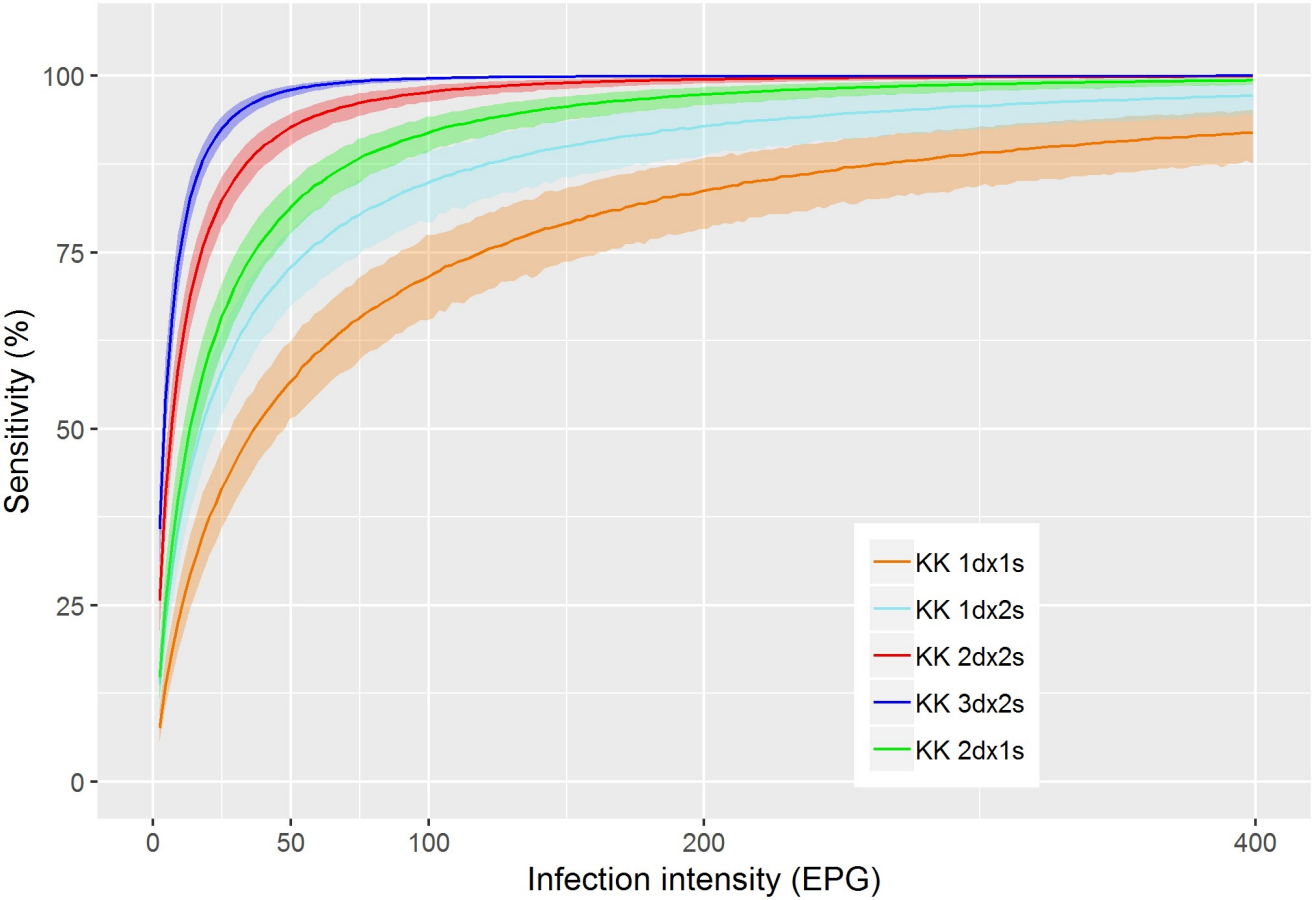
Model-based estimates of the infection intensity-dependent sensitivity of Kato-Katz for various numbers of days (*d*) with one or two samples (*s*).

In Fig 2 the infection intensity-dependent sensitivity of Kato-Katz for one to two slides from one to three samples are shown. Single slide Kato-Katz testing is equivalent to the 2+/3+ POC-CCA proxy with a sensitivity of 60% at 50 EPG, and 70% at 100 EPG. A single slide on two different days clearly outperforms two slides from the same sample. Taking two slides from three different samples leads essentially to perfect sensitivity for infection intensities above 50 EPG but even this extensive sampling scheme does not allow reliable detection of very light infections below 10 EPG.

### Prevalence and mean infection intensity model estimates

Table 4 shows estimates of egg-positive and egg-negative infection prevalence, mean infection intensity of an egg-positive infected individual, and mean egg count in the population. We found a wide variation in the estimates between datasets with egg-positive infection prevalence ranging from 6% to 91%, and egg-negative prevalence varying from 3% to 45%. The mean intensity of an infected individual ranged from 80 EPG to more than 500 EPG and the mean egg count in the population ranged from around 4 EPG to more than 300 EPG.

**Table 4:** Prevalence of egg-positive and egg-negative/antigen-positive cases and infection intensities for each dataset.

Country	Location	Prev. egg-pos. (%) ^1^	Prev. egg-neg. (%) ^2^	Mean infect. (EPG) ^3^	Mean pop. egg-count ^4^ (EPG)
Cameroon	Makenene	91 (85, 96)	3 (0, 8)	221.8 (149.8, 325.8)	75.0 (53.3, 106.2)
Cameroon	Njombe	84 (75, 91)	5 (0, 13)	189.0 (113.4, 308.9)	48.2 (30.9, 72.2)
Cameroon	Yaounde	38 (29, 48)	38 (27, 49)	476.5 (225.2, 986.7)	46.5 (25.1, 81.5)
Côte d’Ivoire	Man	8 (6, 12)	24 (20, 28)	132.2 (65.5, 250.0)	7.6 (3.5, 14.3)
Côte d’Ivoire	1	42 (29, 58)	42 (26, 58)	222.1 (84.9, 515.4)	41.8 (19.4, 80.2)
Côte d’Ivoire	2	50 (31, 70)	23 (4, 41)	245.8 (94.9, 572.1)	65.4 (27.8, 132.6)
Côte d’Ivoire	1	93 (88, 97)	5 (1, 10)	683.5 (535.9, 891.6)	313.9 (250.6, 393.5)
Côte d’Ivoire	2	62 (52, 74)	25 (7, 42)	243.0 (127.5, 442.9)	59.7 (34.7, 97.2)
Côte d’Ivoire	3	51 (39, 63)	8 (0, 28)	868.5 (134.1, 3656.4)	48.4 (17.7, 100.6)
Ethiopia	Harbu	67 (58, 76)	14 (5, 22)	80.5 (59.8, 107.8)	28.2 (21.4, 37.6)
Ethiopia	Jiga	52 (47, 58)	10 (6, 15)	156.7 (125.9, 197.7)	65.0 (50.9, 82.8)
Kenya		30 (27, 34)	45 (41, 49)	119.0 (90.6, 158.8)	11.1 (8.8, 13.9)
Uganda	1	72 (58, 83)	5 (0, 17)	274.3 (143.4, 508.3)	86.5 (46.6, 154.3)
Uganda	2	70 (56, 81)	7 (0, 20)	217.2 (111.5, 398.1)	74.4 (40.0, 126.9)
Uganda	3	38 (25, 53)	27 (14, 39)	167.1 (55.9, 426.8)	27.6 (9.9, 60.9)
Uganda	4	43 (29, 62)	21 (6, 34)	586.6 (247.7, 1,350.2)	70.2 (35.0, 128.9)
Uganda	5	16 (8, 28)	35 (23, 48)	366.3 (136.4, 896.9)	26.4 (9.3, 55.4)
Uganda	Base	12 (9, 16)	13 (9, 17)	303.9 (56.9, 1,347.0)	3.8 (1.6, 8.2)
Uganda	F1	9 (6, 13)	20 (15, 25)	137.5 (53.3, 328.0)	7.5 (3.0, 16.3)
Uganda	Mapping	6 (3, 8)	6 (3, 9)	693.4 (215.0, 2,117.2)	9.0 (3.8, 17.1)

Posterior mean and 95% BCI for all model estimates.

^1^ Estimated prevalence of egg-shedding individuals

^2^ Estimated prevalence of non egg-shedding individuals that harbor worms

^3^ Estimated arithmetic mean infection intensity of an egg-positive infected individual

^4^ Estimated mean egg count in the population

**Fig 3:**
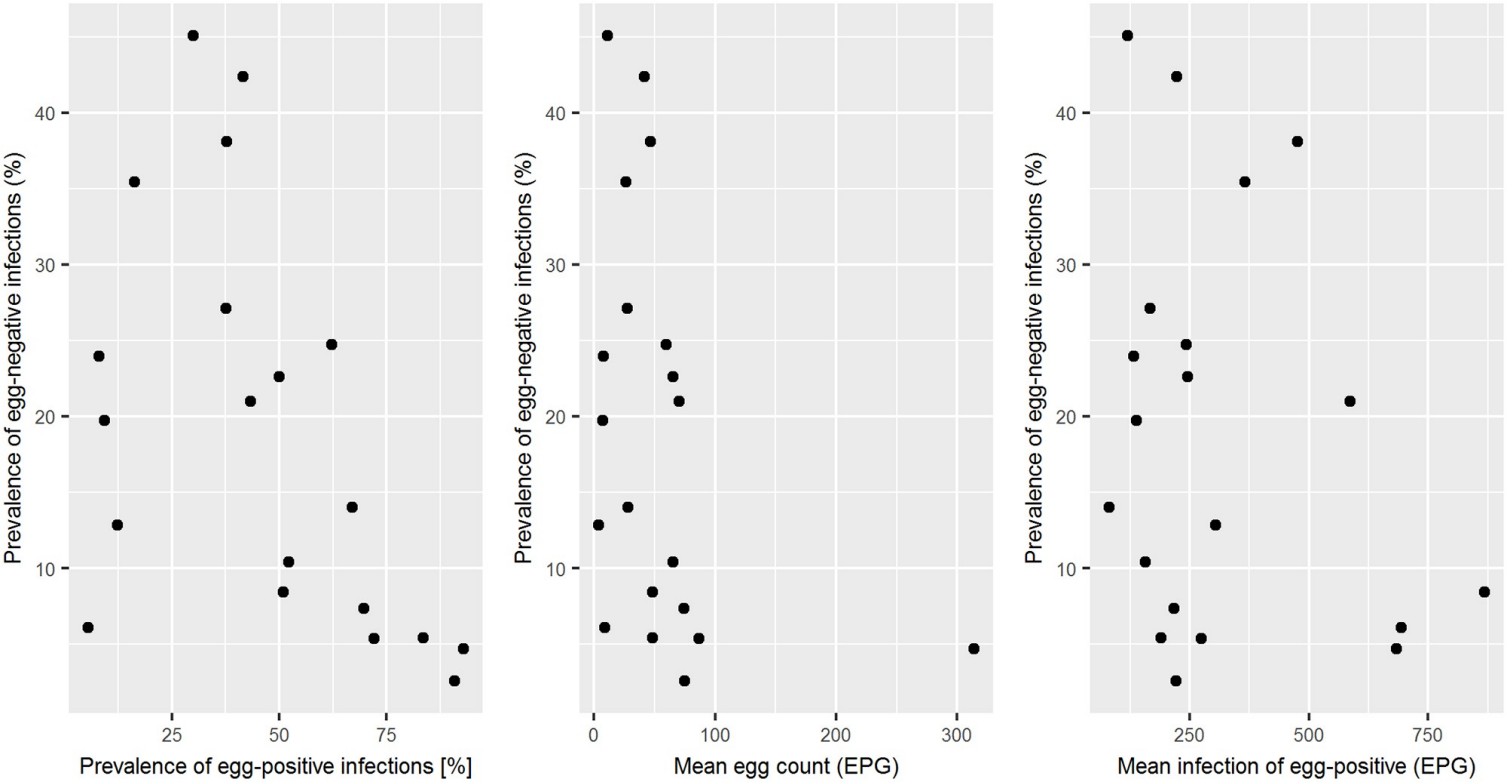
Relation between egg-negative prevalence, egg-positive prevalence, mean egg count in the population, and mean infection intensity of an infected individual.

There was no simple relation between the prevalence of egg-negative and egg-positive infections, the infection intensity of the infected individuals, and the total infection intensity in the population (Fig 3). However, there was evidence of a positive relation between the prevalence of egg-positive and egg-negative infections at low prevalence.

### Relation between Kato-Katz and POC-CCA

**Fig 4:**
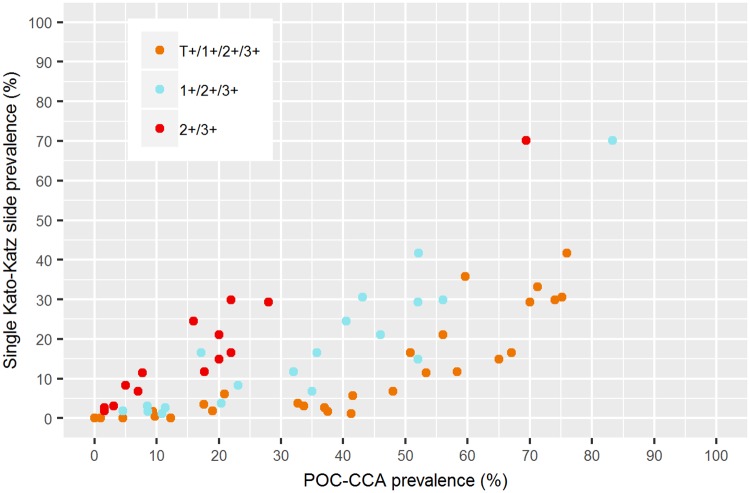
Relation between observed Kato-Katz and POC-CCA prevalence based on the datasets in Tables 1 and 2.

Fig 4 displays the relation between the observed prevalence measured with a single Kato-Katz thick smear and POC-CCA. We observed a clear relation for each of the three proxies of POC-CCA, but there is still insufficient data to give a clear indication of the variability.

**Fig 5:**
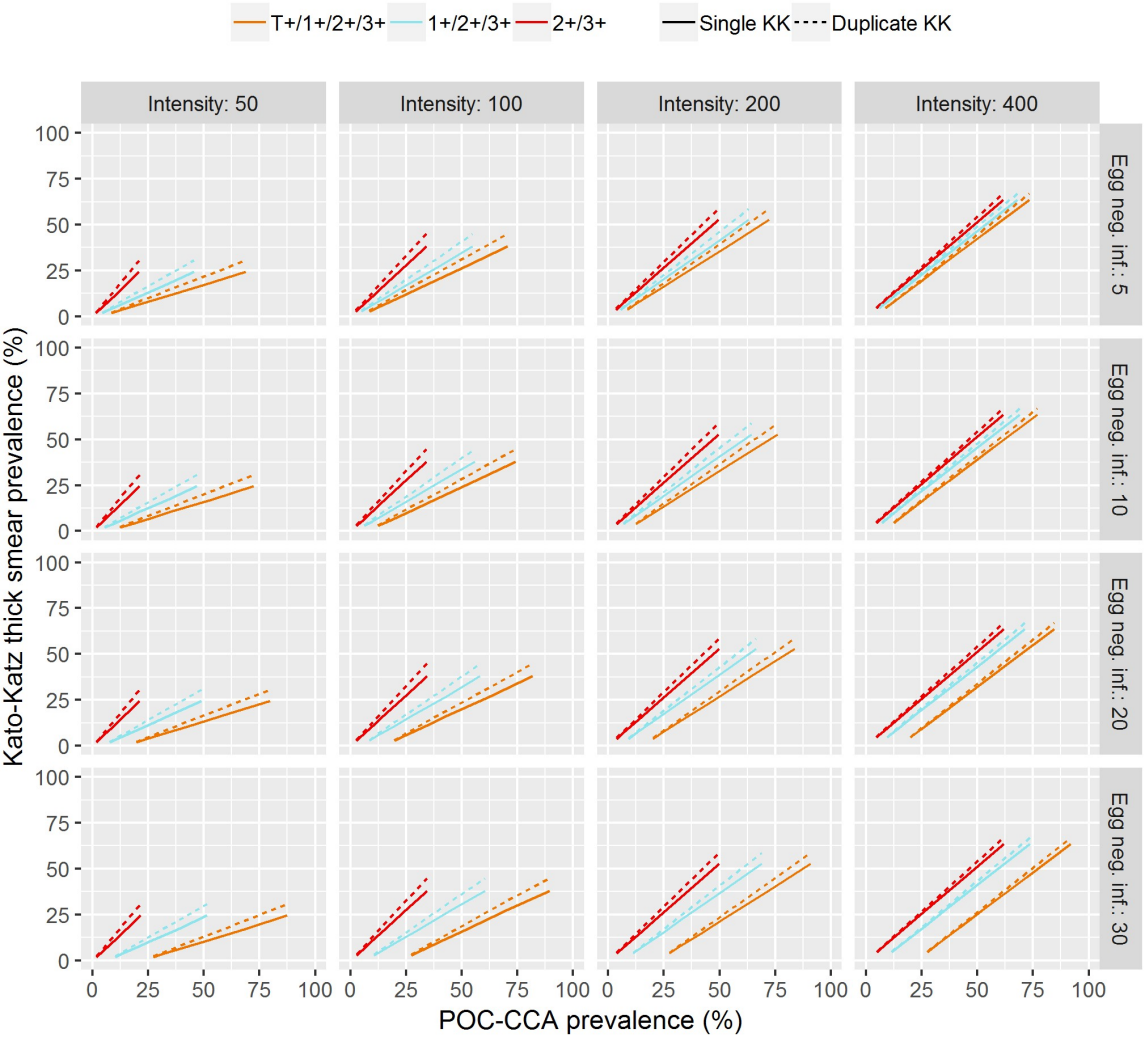
Predictions of the dependence between Kato-Katz and POC-CCA prevalence for various infection intensities and prevalence of egg-negative cases. Infection intensity is given in EPG and egg-negative prevalence in %.

The results of the simulation study (Fig 5) showed the estimated relation between single and duplicate slide Kato-Katz prevalence and POC-CCA prevalence for varying egg-positive prevalence for the 16 combinations with the four infection intensity scenarios on the x-axis and the four scenarios for the prevalence of egg-negative infections on the y-axis.

**Fig 6:**
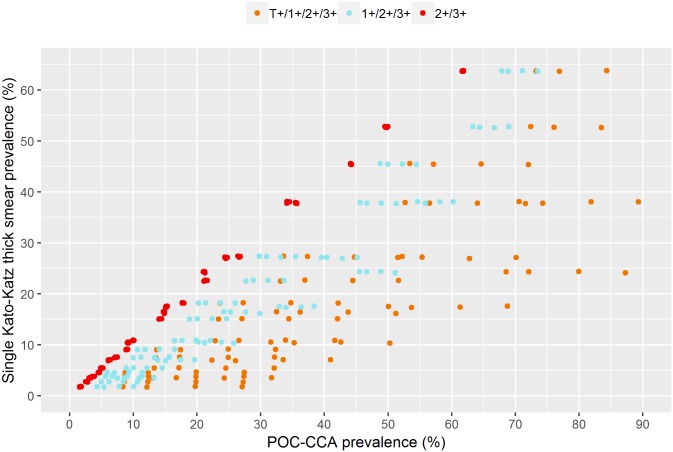
Scatter plot of all results for single slide Kato-Katz from Fig 5.

**Fig 7:**
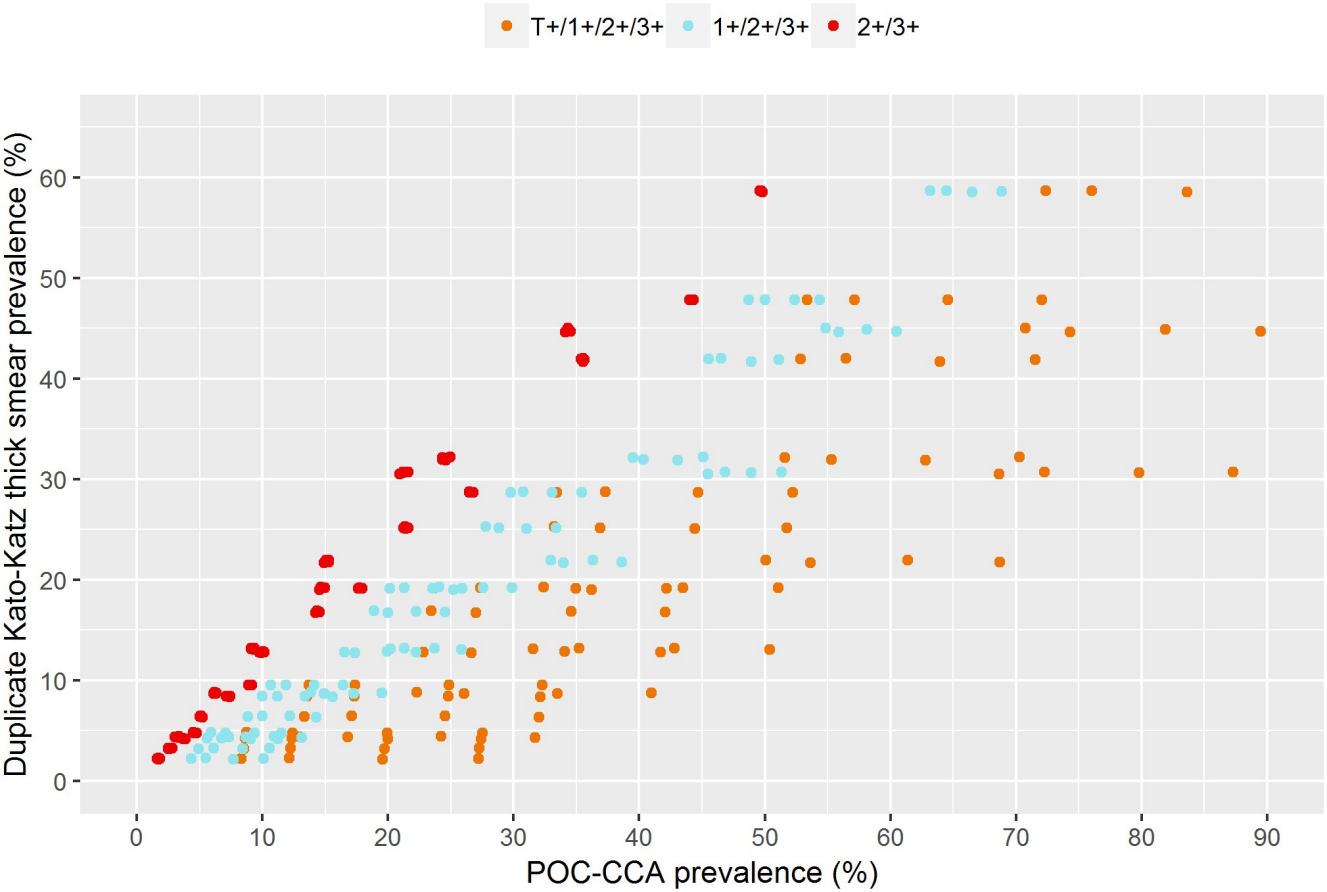
Scatter plot of all results for duplicate slide Kato-Katz from Fig 5.

For trace considered positive, the relation between POC-CCA and Kato-Katz prevalence were dependent on infection intensity and prevalence of egg-negative infections. When Kato-Katz prevalence is zero, the POC-CCA prevalence is determined by the prevalence of egg-negative infections. For low mean infection intensities, there are primarily light infections for which POC-CCA has a higher sensitivity, and hence, the slope of the relation is small. When only 2+ and 3+ readings are considered positive, the relation neither depends on the infection intensity nor on the prevalence of egg-negative infections. When trace is considered negative, there is lower variation in the Kato-Katz/POC-CCA relation. These patterns hold for both single and duplicate slide Kato-Katz with a change in the slope of the relation. Figs 6 and 7 show a scatter plot of prevalence measured by single and duplicate slide Kato-Katz, respectively, and POC-CCA for all simulation scenarios to highlight the variability present in the Kato-Katz/POC-CCA relation for each proxy.

Both the prevalence of egg-negative infections and mean infection intensity are, in practice, unknown when only POC-CCA diagnostics is applied. There is large uncertainty in the estimates for the trace-positive proxy due to the dependence on unknown parameters. For the trace-negative proxy there is weaker but still considerable uncertainty. The 2+ and 3+ proxy shows close to perfect accordance with Kato-Katz and only low uncertainty. Based on the simulation scenarios, Table 5 translates single and duplicate slide Kato-Katz prevalence thresholds into POC-CCA equivalents. For trace considered positive, the variability is large because of the previously mentioned unknown number of egg-negative/antigen-positive individuals. Single slide Kato-Katz and POC-CCA prevalence are identical when 2+ and 3+ proxy is considered. Due to the uncertainty in the Kato-Katz/POC-CCA relation when trace-positive or trace-negative proxies are used, the Kato-Katz prevalence corresponds to a range of POC-CCA prevalence values which are generally higher than the Kato-Katz one. A single slide Kato-Katz prevalence of 10% corresponds to 20–40% POC-CCA prevalence when traces are considered positive and to 15–25% when traces are considered negative. For a duplicate slide Kato-Katz prevalence of 10% the equivalent ranges are 15–40% when traces are considered positive and 10–20% when traces are considered negative.

**Table 5:** Estimated equivalent prevalence of POC-CCA to single and duplicate slide Kato-Katz and suggested equivalent prevalence threshold.

Kato-Katz	POC-CCA T/1+/2+/3+	Suggested threshold	1+/2+/3+	2+/3+
Single				
1%	5-30	10%	3-10%	1%
5%	10-30%	20%	5-15%	5%
10%	20-40%	30%	15-25%	10%
25%	35-70%	50%	30-50%	25%
50%	>75%	75%	>60%	50%
Duplicate				
1%	5-25	10%	3-10%	1%
5%	10-35%	20%	5-15%	5%
10%	15-40%	30%	10-20%	5-10%
25%	30-70%	45%	25-40%	15-25%
50%	>60%	60%	>50%	>40%

When avoiding under-treatment is the priority, conservative thresholds could be defined at the lower end of each range. However, the lower end of the range corresponds to a high mean infection-intensity of an infected individual which is only a realistic scenario in a high prevalence setting. For lower prevalence, a lower mean infection intensity is a more realistic threshold. Hence, we suggest a threshold of 60% POC-CCA to be equivalent to 50% duplicate Kato-Katz, and 30% POC-CCA to be equivalent to 10% Kato-Katz to define treatment categories. Table 6 shows the WHO guidelines for *S. mansoni* from 2013 [8]. In addition to the threshold given for parasitological methods we added the suggested thresholds from Table 5.

**Table 6:** WHO recommended treatment strategy for schistosomiasis mansoni [8] extended with the suggested thresholds for POC-CCA from Table 5.

Category	Prevalence among school-aged children	Action to be taken
High-risk community	≥ 50% by parasitological methods or ≥ 60% by POC-CCA	Treat all school-aged children (enrolled and not enrolled) once a year. Also treat adults considered to be at risk (from special groups or once a year to entire communities living in endemic areas).
Moderate-risk community	≥ 10% by parasitological methods or ≥ 30% by POC-CCA	Treat all school-aged children (enrolled and not enrolled) once every 2 years. Also treat adults considered to be at risk (special groups only).
Low-risk community	≤ 10% by parasitological methods or ≤ 30% by POC-CCA	Treat all school-aged children (enrolled and not enrolled) twice during their schooling age (e.g., once on entry and once on exit). Praziquantel should be available in dispensaries and clinics for treatment of suspected cases.

## Discussion

We established the relation between observed Kato-Katz and POC-CCA prevalence of *S. mansoni* infections using rigorous Bayesian modeling and extensive simulation studies. Moreover, our analysis provided estimates of the infection intensity-dependent sensitivity, the sensitivity for egg-negative/antigen-positive infections, and the specificity of POC-CCA without making use of an artificial ‘gold’ standard.

We found that a duplicate slide Kato-Katz prevalence of 10% corresponds to 15–40% POC-CCA when trace are considered positive and 10–20% POC-CCA when trace are considered negative. The uncertainty in the estimates cannot be eliminated due to the exact value depending on quantities not known by POC-CCA, namely the mean infection intensity in the population and the prevalence of egg-negative but antigen-positive individuals. More accurate estimates can be given using the POC-CCA ≥ 2+ proxy. It shows similar change of the sensitivity with infection intensity and insensitivity to light-intensity infections as single slide Kato-Katz leading to a one-to-one correspondence. However, this stringent proxy misses light intensity infections, as does the Kato-Katz assay.

Previous analyses were unable to give clear recommendations due to various limitations in the models and data used. Kittur et al. (2016) [35] performed a regression analysis on the datasets included in the reviews by Danso-Appiah et al. (2016) [20] and Ochodo et al. (2015) [19] to establish the relation between Kato-Katz and POC-CCA prevalence. The researchers found a clear correlation between the semi-quantitative results of POC-CCA and the egg output measured by Kato-Katz, but the variation in the relation between Kato-Katz prevalence and POC-CCA was too large for predictive use. Prada et al. (2018) [33] applied a Bayesian model on pre- and post-treatment data from a study in Uganda taking into account the infection intensity-dependent sensitivity of POC-CCA. They estimated a specificity of only 85% and determined a relation between Kato-Katz and POC-CCA prevalence for true prevalence above 35%. Caveats of their analysis are as follows. First, the semi-quantitative readings were modeled by a binomial distribution, which is not supported by the process generating the data. Second, POC-CCA positive but egg-negative individuals were defined as negative, thus underestimating specificity. Third, model-based estimates do not represent low endemic settings.

The results presented here revealed that POC-CCA has close to perfect sensitivity for moderate and heavy *S. mansoni* infections (≥ 100 EPG) and a specificity of over 95% regardless of the endemicity. Generally, the uncertainty in the sensitivity estimates is larger than for Kato-Katz due to considerable variation introduced by the lack of a standardized reader. For light infections (1–99 EPG) the sensitivity varies with the proxy used to categorize semi-quantitative POC-CCA readings into a binary result (see Fig 1). The proxy that classifies traces as positives has a very high sensitivity even for infections below 50 EPG and it detects egg-negative/antigen-positive infections with a moderate sensitivity of 75%, while the Kato-Katz technique is unreliable in detecting infections with intensities below 10 EPG even for repeated sampling. The proxy that considers POC-CCA ≥ 1+ as positive has lower sensitivity, but larger variability in the estimates of the infection dependent sensitivity. It follows that POC-CCA has a very high diagnostic sensitivity and specificity at the individual level when traces are included in the positive results.

In two recent systematic reviews and meta-analyses of diagnostic accuracy of POC-CCA, a sensitivity of 90% of POC-CCA was found when trace was included in the positives, which is in agreement with our results [19, 20]. However, the prior analyses assumed Kato-Katz as the diagnostic ‘gold’ standard and did not take into account the dependence of the sensitivity on infection intensity. The former assumption implies that the additional positives of POC-CCA are false-positives. They report a specificity of 55% against single Kato-Katz and a specificity of 66% against duplicate or triplicate Kato-Katz. The increase in specificity when compared to a more accurate ‘gold’ standard indicates that not all additional positives are false-positives and that they underestimate the true specificity. The lack of positive POC-CCA tests in non-endemic regions, the correlation of egg-negative/antigen-positive prevalence with egg-positive prevalence, and our model-based estimates suggest that most of the additional positives detected by POC-CCA are true infections. This interpretation is in agreement with Mwinzi et al. (2015) [28] who showed that the number of POC-CCA-positives but egg-negative decreased after treatment with praziquantel.

### Conclusion

Kato-Katz prevalence can be translated to a range of POC-CCA prevalence. Choosing a single equivalent threshold can be justified for simplicity and applicability but leads to misclassification. A conservative threshold could be chosen at the lower end of the range, which ensures that there is no under-treatment but doing so would also underestimate the true prevalence of disease. Instead, we recommend a more balanced approach suggesting a threshold at the lower end of the range for high prevalence, and a more central value for lower prevalence to reflect the accompanying decrease in mean infection-intensity of the infected population. Therefore, the 10% and 50% duplicate slide Kato-Katz thresholds are to be translated to 30% and 60% POC-CCA, respectively, when traces are considered positive. Additionally, new treatment categories for scenarios close to elimination can be defined for POC-CCA at 10% and 20% roughly corresponding to 1% and 5% Kato-Katz, respectively. For a more accurate translation, which is especially useful when integrating studies based on Kato-Katz and POC-CCA diagnostics into a single analysis, we recommend recording fully semi-quantitative results and using the POC-CCA ≥ 2+ prevalence as equivalent to single slide Kato-Katz.

In suspected low-endemicity settings, we recommend replacing Kato-Katz irrespective of sampling effort with the trace positive proxy of POC-CCA. This is solely based on the diagnostic accuracy determined in this study, while cost-effectiveness shall be evaluated taking into account the specific situation. The test’s sensitivity to infections with none or only erratic egg shedding, which are difficult to detect even by Kato-Katz repeated on multiple days, make it useful for surveillance in settings approaching elimination and for diagnostics on the individual level. The presented evidence suggests that the egg-negative but antigen-positive infections are real infections, and hence, it is conceivable that the current Kato-Katz based estimates for prevalence and morbidity underestimate the reality. Better tools and further studies are needed to determine worm burdens and morbidity associated with egg-negative infections.

## Supporting information

S1 Table Posterior means and 95% BCI of model parameters that determine sensitivity estimates. (PDF)

S2 Table Individual level data from datasets in Group 1. (XLSX)

## References

[1] Colley DG, Bustinduy AL, Secor WE, King CH. Human schistosomiasis. *Lancet*. 2014;383:2253–2264. 10.1016/S0140-6736(13)61949-2 24698483 PMC4672382

[2] Hotez PJ, Alvarado M, Basáñez MG, Bolliger I, Bourne R, Boussinesq M, et al The Global Burden of Disease Study 2010: interpretation and implications for the neglected tropical diseases. *PLoS Negl. Trop. Dis*. 2014;8:e2865 10.1371/journal.pntd.0002865 25058013 PMC4109880

[3] GBD 2016 DALYs and HALE Collaborators. Global, regional, and national disability-adjusted life years (DALYs) for 333 diseases and injuries and healthy life expectancy (HALE) for 195 countries and territories, 1990–2016: a systematic analysis for the Global Burden of Disease Study 2016. *Lancet*. 2017;390:1260–1344. 10.1016/S0140-6736(17)32130-X 28919118 PMC5605707

[4] Cheever EA, Cheever AW, Macedonia JG, Mosimann JE. Kinetics of egg production and egg excretion by *Schistosoma mansoni* and *S. japonicum* in mice infected with a single pair of worms. *Am. J. Trop. Med. Hyg*. 1994;50:281–295. 10.4269/ajtmh.1994.50.281 8147487

[5] van der Werf JM, de Vlas SJ, Brooker S, Looman CWN, Nagelkerke NJD, Habbema JDF, et al Quantification of clinical morbidity associated with schistosome infection in sub-Saharan Africa. *Acta Trop*. 2003;86:125–139. 10.1016/S0001-706X(03)00029-9 12745133

[6] King CH, Dickman K, Tisch DJ. Reassessment of the cost of chronic helmintic infection: a meta-analysis of disability-related outcomes in endemic schistosomiasis. *Lancet*. 2005;365:1561–1569. 10.1016/S0140-6736(05)66457-4 15866310

[7] Ezeamama AE, Bustinduy AL, Nkwata AK, Martinez L, Pabalan N, Boivin NJ, et al Cognitive deficits and educational loss in children with schistosome infection—a systematic review and meta-analysis. *PLoS Negl. Trop. Dis*. 2018;12:e0005524 10.1371/journal.pntd.0005524 29329293 PMC5766129

[8] WHO. Schistosomiasis: progress report 2001–2011 and strategic plan 2012–2020. World Health Organization, Geneva, 2013.

[9] Lo NC, Addiss DG, Hotez PJ, King CH, Stothard JR, Evans DS, et al A call to strengthen the global strategy against schistosomiasis and soil-transmitted helminthiasis: the time is now. *Lancet Infect. Dis*. 2017;17:e64–69. 10.1016/S1473-3099(16)30535-7 27914852 PMC5280090

[10] Stothard JR, Stanton MC, Bustinduy AL, Sousa-Figueiredo JC, van Dam GJ, Betson M, et al Diagnostics for schistosomiasis in Africa and Arabia: a review of present options in control and future needs for elimination. *Parasitology*. 2014;141:1947–1961. 25158604 10.1017/S0031182014001152

[11] Utzinger J, Becker SL, van Lieshout L, van Dam G, Knopp S. New diagnostic tools in schistosomiasis. *Clin. Microbiol. Infect*. 2015;21:529–542. 10.1016/j.cmi.2015.03.014 25843503

[12] Knowles SCL, Sturrock HJW, Turner H, Whitton JM, Gower CM, Jemu S, et al Optimising cluster survey design for planning schistosomiasis preventative chemotherapy. *PLoS Negl. Trop. Dis*. 2017;11:e0005599 10.1371/journal.pntd.0005599 28552961 PMC5464666

[13] Katz N, Chaves A, Pellegrino J. A simple device for quantitative stool thick-smear technique in schistosomiasis mansoni. *Rev. Inst. Med. Trop. São Paulo*. 1972;14:397–400. 4675644

[14] Montresor A, Crompton DWT, Hall A, Bundy DAP, Savioli L. Guidelines for the evaluation of soil-transmitted helminthiasis and schistosomiasis at community level. World Health Organization, Geneva, 1998.

[15] King CH. It’s time to dispel the myth of “asymptomatic” schistosomiasis. *PLoS Negl. Trop. Dis*. 2015;9:e0003504 10.1371/journal.pntd.0003504 25695740 PMC4335065

[16] Bärenbold O, Raso G, Coulibaly JT, N’Goran EK, Utzinger J, Vounatsou P. Estimating sensitivity of the Kato-Katz technique for the diagnosis of *Schistosoma mansoni* and hookworm in relation to infection intensity. *PLoS Negl. Trop. Dis*. 2017;11:e0005953 10.1371/journal.pntd.0005953 28976979 PMC5643140

[17] Booth M, Vounatsou P, N’Goran EK, Tanner M, Utzinger J. The influence of sampling effort and the performance of the Kato-Katz technique in diagnosing *Schistosoma mansoni* and hookworm co-infections in rural Côte d’Ivoire. *Parasitology*. 2003;127:525–531. 14700188 10.1017/s0031182003004128

[18] van Lieshout L, Polderman AM, Deelder AM. Immunodiagnosis of schistosomiasis by determination of the circulating antigens CAA and CCA, in particular in indiciduals with recent or light infections *Acta Trop*. 2000;77:69–80. 10.1016/S0001-706X(00)00115-7 10996122

[19] Ochodo EA, Gopalakrishna G, Spek B, Reitsma JB, van Lieshout L, Polman K, et al Circulating antigen tests and urine reagent strips for diagnosis of active schistosomiasis in endemic areas. *Cochrane Database Syst. Rev*. 2015;Issue 3:CD009579 10.1002/14651858.CD009579.pub2 25758180 PMC4455231

[20] Danso-Appiah A, Minton J, Boamah D, Otchere J, Asmah RH, Rodgers M, et al Accuracy of point-of-care testing for circulatory cathodic antigen in the detection of schistosome infection: systematic review and meta-analysis. *Bull. World Health Organ*. 2016;94:522–533. 10.2471/BLT.15.158741 27429491 PMC4933137

[21] Colley DG, Binder S, Campbell C, King CH, Tchuem-Tchuenté LA, N’Goran EK, et al A five-country evaluation of a point-of-care circulating cathodic antigen urine assay for the prevalence of *Schistosoma mansoni*. *Am. J. Trop. Med. Hyg*. 2013;88:426–432. 10.4269/ajtmh.12-0639 23339198 PMC3592520

[22] Coulibaly JT, Knopp S, N’Guessan NA, Silué KD, Fürst T, Lohourignon LK, et al Accuracy of urine circulating cathodic antigen (CCA) test for *Schistosoma mansoni* diagnosis in different settings of Côte d’Ivoire. *PLoS Negl. Trop. Dis*. 2011;5:e1384 10.1371/journal.pntd.0001384 22132246 PMC3222626

[23] Shane HL, Verani JR, Abudho B, Montgomery SP, Blackstock AJ, Mwinzi P, et al Evaluation of urine CCA assays for detection of *Schistosoma mansoni* infection in western Kenya. *PLoS Negl. Trop. Dis*. 2011;5:e951 10.1371/journal.pntd.0000951 21283613 PMC3026766

[24] Tchuem-Tchuenté LA, Fouodo CJK, Ngassam RIK, Sumo L, Noumedem CD, Kenfack CM, et al Evaluation of circulating cathodic antigen (CCA) urine-tests for diagnosis of *Schistosoma mansoni* infection in Cameroon. *PLoS Negl. Trop. Dis*. 2012;6:e1758 10.1371/journal.pntd.0001758 22860148 PMC3409114

[25] Assaré RK, Tra MBI, Ouattara M, Hürlimann E, Coulibaly JT, N’Goran EK, et al Sensitivity of the Point-of-Care Circulating Cathodic Antigen urine cassette test for diagnosis of *Schistosoma mansoni* in low-endimicity settings in Côte d’Ivoire. *Am. J. Trop. Med. Hyg*. 2018 10.4269/ajtmh.18-0550 30277203 PMC6283482

[26] Coulibaly JT, N’Gbesso YK, Knopp S, N’Guessan NA, Silué KD, van Dam G, et al Accuracy of urine circulating cathodic antigen test for the diagnosis of *Schistosoma mansoni* in preschool-aged children before and after treatment. *PLoS Negl. Trop. Dis*. 2013;7:e2109 10.1371/journal.pntd.0002109 23556011 PMC3605147

[27] Erko B, Medhin G, Teklehaymanot T, Degarege A, Legesse M. Evaluation of urine-circulating cathodic antigen (urine-CCA) cassette test for the detection of *Schistosoma mansoni* infection in areas of moderate prevalence in Ethiopia. *Trop. Med. Int. Health*. 2013;18:1029–1035. 10.1111/tmi.12117 23590255

[28] Adriko M, Standley CJ, Tinkitina B, Tukahebwa E, Fenwick A, Fleming FM, et al Evaluation of circulating cathodic antigen (CCA) urine-cassette assay as a survey tool for schistosomiasis mansoni in different transmission settings within Bugiri district, Uganda. *Acta Trop*.2014;136:50–57. 10.1016/j.actatropica.2014.04.001 24727052

[29] Haggag AA, Rabiee A, Abd Elaziz KM, Gabrielli AF, Abdel Hay R, Ramzy RMR. Mapping of *Schistosoma mansoni* in the Nile delta, Egypt: Assessment of the prevalence by the circulating cathodic antigen urine assay. *Acta Trop*. 2017;167:9–17. 10.1016/j.actatropica.2016.11.038 27965144

[30] Ortu G, Ndayishimiye O, Clements M, Kayugi D, Campbell CH, Lamine MS, et al Countrywide reassessment of *Schistosoma mansoni* infection in Burundi using a urine-circulating cathodic antigen rapid test: informing the national control program. *Am. J. Trop. Med. Hyg*. 2017;96:664–673. 10.4269/ajtmh.16-0671 28115675 PMC5361543

[28] Mwinzi PNM, Kittur N, Ochola E, Cooper PJ, Campbell CH, King CH, et al Additional evaluation of the point-of-contact circulating cathodic antigen assay for *Schistosoma mansoni* infection. *Front. Public Health*. 2015;3:48 10.3389/fpubh.2015.00048 25853117 PMC4365547

[32] Bottomley C, Isham V, Vivas-Martinez S, Kuesel AC, Attah SK, Opoku NO, et al Modelling neglected tropical diseases diagnostics: the sensitivity of skin snips for *Onchocerca volvulus* in near elimination and surveillance settings. *Parasit. Vectors*. 2016;9:343 10.1186/s13071-016-1605-3 27301567 PMC4908809

[33] Prada JM, Touloupou P, Adriko M, Tukahebwa E, Lamberton P, Hollingsworth TD. Understanding the relationship between egg- and antigen-based diagnostics of *Schistosoma mansoni* infection pre- and post-treatment in Uganda. *Parasit. Vectors*. 2018;11:21 10.1186/s13071-017-2580-z 29310695 PMC5759883

[34] Carpenter B, Gelman A, Hoffman M, Lee L, Goodrich B, Betancourt M, et al Stan: a probabilistic programming language. *J. Stat. Softw*. 2016. 10.18637/jss.v076.i01 PMC9788645 36568334

[35] Kittur N, Castleman JD, Campbell CH, King CH, Colley DG. Comparison of *Schistosoma mansoni* prevalence and intensity of infection, as determined by the circulating cathodic antigen urine assay or by the Kato-Katz fecal assay: a systematic review. *Am. J. Trop. Med. Hyg*. 2016;94:605–610. 10.4269/ajtmh.15-0725 26755565 PMC4775897

