# Supplementary material for: Translating preventive chemotherapy prevalence thresholds for Schistosoma mansoni from the Kato-Katz technique into the point-of-care circulating cathodic antigen diagnostic test
Source: PLoS Negl Trop Dis. 2018 Dec 14;12(12):e0006941. doi: 10.1371/journal.pntd.0006941 (PMC6310297; doi:10.1371/journal.pntd.0006941)
Supplement: S1 Table — (PDF) [file pntd.0006941.s001.pdf]

Supporting information

S1 Table. Posterior means and 95% BCI of model parameters that determine sensitivity estimates.

	Posterior mean	95% BCI
**Kato-Katz**		
α	0.07	0.04–0.11
γ_2	5.34	3.07–10.10
σ	1.28	1.08–1.49
**T/1+/2+/3+**		
a_0	1.10	0.62–1.67
a_1	3.64	1.97–6.25
a_2	5.58	4.55–6.87
**1+/2+/3+**		
a_0	-1.20	-1.95– -0.42
a_1	3.75	1.90–6.59
a_2	3.69	2.78–4.68
**2+/3+**		
a_0	-4.46	-5.16– -3.74
a_1	4.34	3.47–5.32
a_2	3.72	2.71–4.53

Table A: Posterior means and 95% BCI of model parameters that determine sensitivity of POC-CCA and Kato-Katz
